# Strategies in times of crisis—insights into the benthic foraminiferal record of the Palaeocene–Eocene Thermal Maximum

**DOI:** 10.1098/rsta.2017.0328

**Published:** 2018-09-03

**Authors:** Daniela N. Schmidt, Ellen Thomas, Elisabeth Authier, David Saunders, Andy Ridgwell

**Affiliations:** 1 School of Earth Sciences, University of Bristol, Wills Memorial Building, Bristol BS8 1RJ, UK; 2 Department of Geology and Geophysics, Yale University, 210 Whitney Avenue, New Haven, CT 06511, USA; 3 School of Geographical Science, University of Bristol, University Road, Bristol BS8 1SS, UK; 4 Department of Earth Sciences, Geology Building, University of California, Riverside, 900 University Avenue, Riverside, CA 92521, USA

**Keywords:** benthic foraminifers, Palaeocene–Eocene Thermal Maximum, µ-computed tomography, ocean drilling programme, climate change, development

## Abstract

Climate change is predicted to alter temperature, carbonate chemistry and oxygen availability in the oceans, which will affect individuals, populations and ecosystems. We use the fossil record of benthic foraminifers to assess developmental impacts in response to environmental changes during the Palaeocene–Eocene Thermal Maximum (PETM). Using an unprecedented number of µ-computed tomography scans, we determine the size of the proloculus (first chamber), the number of chambers and the final size of two benthic foraminiferal species which survived the extinction at sites 690 (Atlantic sector, Southern Ocean, palaeodepth 1900 m), 1210 (central equatorial Pacific, palaeodepth 2100 m) and 1135 (Indian Ocean sector, Southern Ocean, palaeodepth 600–1000 m). The population at the shallowest site, 1135, does not show a clear response to the PETM, whereas those at the other sites record reductions in diameter or proloculus size. Temperature was similar at all sites, thus it is not likely to be the reason for differences between sites. At site 1210, small size coincided with higher chamber numbers during the peak event, and may have been caused by a combination of low carbonate ion concentrations and low food supply. Dwarfing at site 690 occurred at lower chamber numbers, and may have been caused by decreasing carbonate saturation at sufficient food levels to reproduce. Proloculus size varied strongly between sites and through time, suggesting a large influence of environment on both microspheric and megalospheric forms without clear bimodality. The effect of the environmental changes during the PETM was more pronounced at deeper sites, possibly implicating carbonate saturation.

This article is part of a discussion meeting issue ‘Hyperthermals: rapid and extreme global warming in our geological past’.

## 1. Introduction

The anthropogenic increase in atmospheric CO_2_ impacts the physical, chemical and biological properties of the ocean [1]. In high-end scenarios, the rise in CO_2_ is modelled to result in a further increase in global mean surface temperatures by 2.6–4.8°C [2], and a lowering of the pH by an additional 0.3–0.4 units by 2100 [3]. At depth, warming is projected to be largest in the Southern Ocean [2]. Changes in these environmental parameters are projected to impact marine species, as well as their interaction with their environment and with other species (e.g. [1,4]). Warming directly affects species by increasing the rate of metabolic processes such as feeding and growth, which are, however, limited by food availability. Species which cannot regulate their temperature may be more strongly impacted [5], though they show a wide range of species-specific responses [5]. The response to ocean acidification includes reduced fertilization, decreases in larval and adult growth rates, reduced calcification and increased mortality [1]. However, some species are able to upregulate their internal pH as adults, and may continue to grow [6]. Even during acidification, high food availability may provide sufficient energy to sustain physiological processes in juvenile bivalves [7], but the effects of multiple impactors need further studies.

Environmental factors influence an organism through developmental plasticity, thereby providing a target on which evolution can act to produce novel, potentially adaptive, phenotypes [8]. Multi-generational experiments assessing the potential for acclimatization [9,10] suggest that adaptive evolution can help to maintain physiological processes otherwise strongly impacted by climate change. Such adaptation could facilitate survival during rapid climate change. Therefore, it is paramount to determine the effects of environmental change not just on the morphology of adult individuals, but across ontogeny.

The fossil record documents natural climate change and variability as well as preserving some species exposed to these environmental changes [11]. The Palaeocene–Eocene Thermal Maximum (PETM), 56 Ma, is the best studied hyperthermal event in the geological record, with significant warming over a few thousand years [12], global changes in carbonate chemistry [13,14], a reduction in oxygen concentrations in the oceans [15] and in surface and deep waters [16] and the resulting biotic responses [17,18]. Foraminifers have an excellent ocean-wide distribution and preservation potential, making it possible to quantify the impact of climate change in the geological record. Benthic foraminifers live in the dark, cold, deep ocean, at comparably stable physical environmental conditions, and, like metazoans in the same environment [19], display a high species diversity [20]. The impact of climate change on benthic deep-sea organisms is significantly less well understood than is the case for shallow water organisms, mainly because of the difficulties in collecting them from their habitat and successfully culturing them, where necessary at *in situ* pressures [21]. Traditionally, impacts of climate change in the fossil record have been assessed in terms of relative or absolute abundance of species, and their origination and extinction. Such data show that the PETM resulted in a significant extinction of benthic foraminifera [21] and a transient faunal turnover [22], as well as migration to higher latitudes in planktic species [23,24]. Experimentally, it has been shown that foraminifers are able to control their calcification [25] and, using novel tomographic methodologies [22], unexpectedly increased calcification during the PETM at least at some locations [26].

Many foraminifers grow by sequentially adding chambers and hence preserve their entire ontogeny in their morphology [27], which can be revealed by tomography [28,29], a technique using X-rays to reveal the internal features of objects. Some benthic foraminifers can alternate between sexual and asexual reproduction, as recorded in the size of the first chamber (proloculus) [30]: the asexually produced, haploid generation generally has a large proloculus and is called megalospheric, whereas the sexually produced diploid generation usually has a smaller proloculus and is called microspheric [31]. Little is known to date about morphological plasticity within the megalospheric and microspheric stages, and the potential link to environmental variability. Body size is a central feature of all organisms, reflecting their physiology, ecology and evolutionary history [32], including metabolism, respiration, calcification and—in the case of foraminifers—number of offspring, which is related to terminal size [33,34]. In some deep-sea environments, foraminifera have a short life span (less than 1 year) because seasonal food flux triggers rapid reproduction, whereas species which do not access the fluctuating food supply have a life cycle of greater than 2 years [35].

Here, we use three-dimensional µ-computed tomography imaging to collect information on proloculus size, number of chambers and final size of two species of benthic foraminifera which survived the extinction at the PETM, at three locations, to quantify response by benthic foraminifers to the climatic and environmental perturbation. Based on our understanding of climate change impacts, competing stressors can impact growth in foraminifers. For example, dwarfing is a common physiological response to environmental stress (e.g. low oxygen, low carbonate saturation [36]). Alternatively, changes in development caused by delayed reproduction in challenging environments have been suggested to lead to larger individuals with more chambers [37]. Increased stress should favour sexual reproduction, but it is not clear whether this leads to earlier maturity and fewer chambers, or if indeed size and number of chambers are related at all.

## 2. Material and methods

### (a) Materials

**Figure 1.**
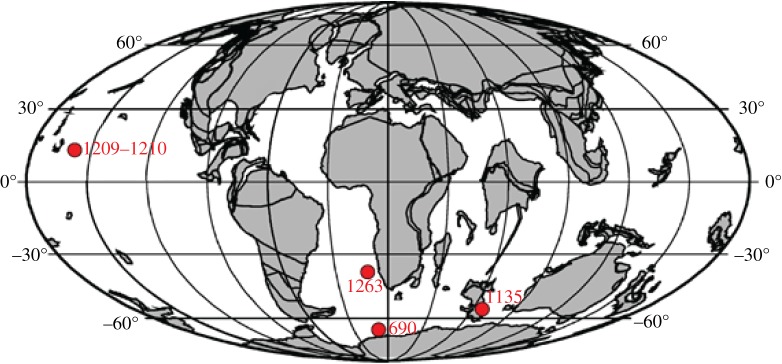
Palaeogeographic map of the locations of the study and the study site of Foster *et al*. [26]. Map generated using ODSN Paleomap (http://www.odsn.de/odsn/services/paleomap/paleomap.html; accessed February 2017). (Online version in colour.)

Samples from three ocean drilling programme (ODP) sites were analysed to compare trends across the PETM in different environmental settings (figure 1). Site 690 (Maud Rise) is in the Atlantic sector of the Southern Ocean, at a palaeodepth of 1900 m [38]; site 1210 (Shatsky Rise) is in the central equatorial Pacific at a palaeodepth of 2100 m and site 1209 is at a palaeodepth of approximately 1900 m [39]; and site 1135 (Kerguelen Plateau) is in the Indian Ocean sector of the Southern Ocean at a palaeodepth of 600–1000 m [40]. The age model for site 690 follows Röhl *et al*. [41], for site 1135 Jiang & Wise [42], for site 1210 Westerhold *et al*. [43] and for site 1209 Westerhold *et al.* [44]. Samples were chosen relative to the carbon isotope excursion (CIE) to represent pre-CIE (before PETM), core CIE, recovery and post-CIE (after PETM), influenced by the availability of benthic species, which is affected by the extinction event. At site 690, *Nuttallides truempyi* is absent in the lowermost peak CIE.

Carbon isotopes for site 1135 were measured at the University of California Santa Cruz SIL facilities at Santa Cruz, CA, USA. From all samples, 10–15 *N. truempyi* specimens were measured. All values are reported relative to the Vienna Pee Dee Belemnite (VPDB) standard. Analytical precision based on replicate analyses of in-house standard Carrara Marble and NBS-19 averages 0.04% (1 s) for *δ*^13^C and 0.07% (1 s) for *δ*^18^O. Carbon isotopes for site 1210 are from [39] and for site 690 from [38].

### (b) Environmental background and model information

Bottom water temperatures at all sites were comparable before the CIE, with similar warming in response to the carbon injection [45–47]. Palaeo-productivity is notoriously difficult to quantify, but, in general, lowered productivity is expected in more stratified waters (preventing nutrient upwelling) during warmer climates [40]. Barium accumulation data are interpreted as indicating that export production was low at all our locations, with the lowest values in the Pacific gyre [48] and higher values in the Southern Ocean [49]. The data also suggest that export production increased at site 690 during the CIE, but did not change in the Pacific gyre. The data on Ba accumulation, however, reflect not directly primary productivity, but remineralization at deeper levels [48]. Remineralization is projected to increase at higher temperatures, even at constant productivity [50,51]. The general picture was corroborated by a recent review of combined data and modelling results [15], which suggested increased oligotrophy at Shatsky Rise, and generally oligotrophic conditions but with short-term increases in the food supply at the onset of the CIE for the Southern Ocean sites.

Based on redox-sensitive elements in the sediments, Kerguelen Plateau may have seen suboxic conditions during the PETM [49,52], whereas at site 690 suboxic conditions may have appeared 90–140 kyr after the onset of the CIE [52,53]. The information for Shatsky Rise is ambiguous, with trace elements suggesting oxic conditions throughout [52].

There are no direct measurements of carbonate ion changes in the deep ocean published to date. Surface ocean pH reconstructions based on boron isotopes in the South [14] and North [54] Atlantic suggest changes around 0.3 pH units. Modelled changes in carbonate saturation in the deep ocean strongly depend on the rate and amount of carbon input [13], and suggest a global average pH change in the deep ocean below 2 km of 0.25 pH units.

**Figure 2.**
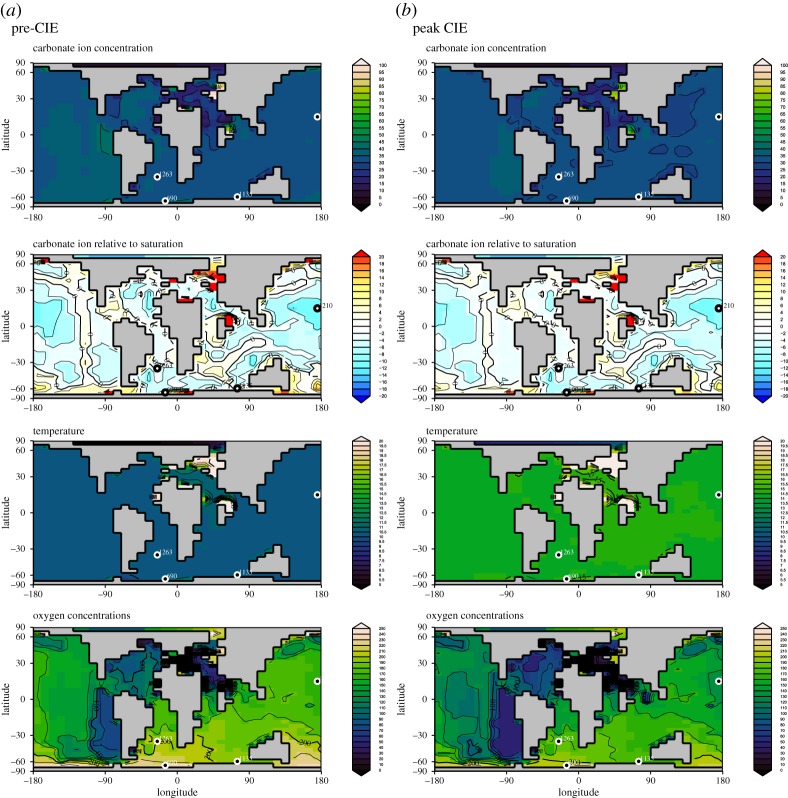
Environmental reconstructions derived from cGENIE for bottom water conditions at each location. From top to bottom carbonate ion concentration (µmol kg^−1^), carbonate ion concentration relative to saturation with positive values indicating locally saturated conditions, temperature (°C) and oxygen concentration (µmol kg^−1^). (*a*) Onset of the CIE; (*b*) 30 kyr into the CIE. (Online version in colour.)

To provide a larger granularity, we used cGENIE to estimate environmental change (table 1 and figure 2). The model parametrization and details of the model are as in Gutjahr *et al*. [54]. We used the early Eocene configuration [55] with the terrestrial weathering feedback. The time points are from the very start of the onset and peak *δ*^13^C minimum time at 30 kyr after the onset of the CIE. We first spun up the model under late Palaeocene boundary conditions, choosing an open-system run time of 200 kyr in order to bring the *δ*^13^C cycle into balance. The model temperatures and warming agree well with the proxy data. Carbonate ion concentrations at the onset and peak of the CIE are low and close to undersaturation at all sites, but especially at site 1210.

**Table 1.** Reconstructed environmental changes using cGENIE for bottom water conditions at each location representing start of the CIE and less than 30 kyr after the onset of the event. ΔCarb is the carbonate ion concentration relative to saturation with positive values indicating locally saturated conditions.

site	CO_3_^2−^(µmol kg^−1^)	ΔCarb (µmol kg^−1^)	*T* (°C)	oxygen (µmol kg^−1^)
start CIE				
690	39.5	14.4	11.0	238.1
1135	29.9	8.1	10.9	179.7
1210	32.6	5.1	10.4	181.9
peak CIE				
690	34.6	10.6	15.0	220.0
1135	28.1	7.1	14.8	161.3
1210	29.9	3.7	14.4	163.6
difference start to peak				
690	−4.8	−3.8	4.0	−18.1
1135	−1.8	−1.0	3.9	−18.4
1210	−2.6	−1.4	4.0	−18.3

### (c) µ-Computed tomography

In total, we scanned and analysed 387 specimens. We focused on two species: the extant shallow infaunal [46,56,57] *Oridorsalis umbonatus* and the extinct, probably epifaunal *N. truempyi*. The latter's descendent, *Nuttallides umbonifera*, lives epifaunally, and is adapted to deep-water environments with carbonate undersaturation [58] and highly oligotrophic conditions [59], thus is common at great depths. All *N. truempyi* and *O. umbonatus* were picked from the greater than 63 residues of the sample of each time slice, with the majority containing greater than 10 individuals (min. 6 to max. 31 individuals). For some specimens, overall size or even chamber number could be determined, but measuring proloculus size was impossible due to internal dissolution. *Oridorsalis umbonatus* was much less common in the studied samples (except for site 1135), and specimens were commonly not sufficiently well preserved to determine the chamber number (or proloculus size) reliably at site 1210.

The specimens were scanned using a Nikon XT H 225 ST CT scanner at 120 kV, a 58 µA current and an exposure time of 0.5 s. Each scan project consisted of 3141 projections, resulting in between 300 and 800 images (voxel size of 2.31 µm), which encompassed all the foraminifera of a single time slice. Slice data from the scans were imported into the three-dimensional visualization software Avizo (Mercury Computer Systems Ltd, Chelmsford, MA, USA, www.tgs.com) to allow investigation of the internal features. As pixels are assigned a grey-scale value to represent the different X-ray attenuation properties of the materials making up the sample, the calcite test of the foraminiferal specimens could be isolated from the mount and any residual sediment infilling.

Final test diameter, number of chambers and size of proloculus were measured for individual specimens following Foster *et al*. [26], in samples from before the CIE, in the core of the CIE, in the recovery interval and after the CIE (table 2).

**Table 2.** Mean values and standard error (s.e.) for proloculus volume, number (no.) of chambers and diameter for *Nuttalides truempyi* (NT) and *O. umbonatus* (OU). Ages (kyr) are relative to onset of CIE; see Material and methods for references for the age models.

core section depth	age (kyr)	species	proloculus volume (µm^3^)	s.e.	no. chambers	s.e.	diameter (µm)	s.e.
1135-25R-3-2	646	NT	11 322	2563.8	23.9	1.3	376	18.1
1135_25R4_56	140	NT	3845	440.4	23.8	0.7	310	9.6
1135_25R4_92	23	NT	8038	1141.4	20.9	0.6	293	9.6
1135_25R4_105	−23	NT	10 870	3106.4	22.0	1.0	343	12.3
1135_25R4_110	−35	NT	19 137	5101.1	19.3	0.8	287	12.7
1135_26R1_90	−1211	NT	9439	2063.2	22.3	0.7	329	14.7
1209B_21H6_100	967	NT	14 720	1903.0	19.9	0.6	259	8.0
1210_20H_6_19	472	NT	18 567	4363.8	20.0	0.9	325	21.8
1210_20H_6_35	205	NT	12 405	4701.6	20.4	1.3	276	26.0
1210_20H_6_46	75	NT	2817	1137.5	22.3	1.2	235	11.9
1210_20H_6_50	23	NT	1464	291.2	24.5	2.3	259	28.2
1210_20H_6_55	−62	NT	1890	302.3	22.4	0.9	241	13.3
1210_20H_6_62	−188	NT	4759	570.8	21.5	0.6	251	5.7
690B-17H_3_74	655	NT	12 517	3387.9	20.4	0.9	304	12.9
690B-19H-1-114	125	NT	11 197	2534.9	23.4	1.2	272	9.2
690B-19H-2-77	92	NT	8650	2261.9	19.1	1.3	229	14.1
690B-19H3-15-16	40	NT	5267	1816.4	21.8	0.9	222	19.8
690B-19H3-43-44	22	NT	9668	1070.7	19.7	1.0	231	12.7
690B-19H-3-86	−6	NT	6673	1851.2	20.6	1.6	293	28.3
690B-19H-3-118	−19	NT	7686	2434.5	22.8	0.9	334	8.6
1135-25R-3-2	646	OU	7820	2065.90	19.8	1.23	313	19.67
1135_25R4_56	140	OU	26 454	6245.03	21.3	1.41	362	15.68
1135_25R4_92	23	OU	22 429	3382.42	16.4	0.58	287	7.57
1135_25R4_105	−23	OU	31 531	5245.29	17.0	0.41	363	43.73
1135_25R4_110	−35	OU	28 902	3198.73	16.0	0.69	290	13.47
1135_26R1_90	−1211	OU	42 662	0.00	17.0	0.00	327	0.00
690B-17H_3_74	655	OU	24 995	11267.31	17.6	2.58	390	11.00
690B-19H-1-114	125	OU	31 232	10281.83	15.8	2.14	257	24.06
690B-19H-2-77	92	OU	10 424	1784.68	22.3	1.20	276	12.22
690B-19H-3-118	−19	OU	23 648	5935.00	19.5	1.50	313	29.18

## 3. Results

### (a) Chamber number

**Figure 3.**
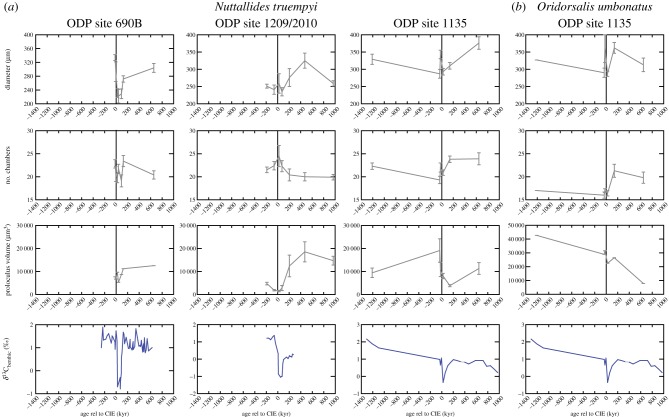
Mean population values for number of chambers (top), final diameter (middle) and proloculus size for *Nuttallides truempyi* at sites 690, 1210/1209 and 1135 (*a*) and for *Oridorsalis umbonatus* at site 1135 (*b*). The error bars represent the s.e. of the mean. The CIE is for reference at the bottom. For references for the carbon isotopes, see Material and methods. (Online version in colour.)

Chamber numbers for *N. truempyi* are highly variable in all samples, though the averages in the populations are surprisingly stable (table 2), ranging at site 1135 from 19 to 23 with an average of 22; at site 1210 from 20 to 25 with an average of 22; and at site 690 from 19 to 23, with an average of 21 (figure 3*a*). At the last site, the population contains some specimens with fewer chambers (minimum 13). There is no clear trend in the number of chambers associated with the core CIE: chamber number increases at site 1210, decreases at site 690 (with trends starting in the sample prior to the CIE, approx. 9 kyr) with a brief recovery followed by a second low, and shows no systematic change at the shallowest site 1135. Average chamber number in the population of *O. umbonatus* ranges between 17 and 22.

### (b) Size

For *N. truempyi*, the ranges of test diameters are similar at all sites, from 192 to 474 µm with mean values highest at site 1135 and lowest at site 1210 (figure 3*b*). Within the peak CIE, sizes at sites 690 are lower than below or above the event with a reduction from 334 µm prior to the event to 222 µm 39.4 kyr below the CIE; note that the size starts to decrease in the sample 9 kyr before the large change in carbon isotopes. By contrast, at the shallower site 1135, large sizes are present throughout.

Analogous to *N. truempyi*, *O. umbonatus* sizes are largest at the shallowest site 1135 (mean of 348 µm with little variation) and smallest at site 1210 (mean 278 µm; see electronic supplementary material site, Figure SI1). The average diameter in the population decreased by 100 µm at site 1210 and by 130 µm at site 690 (table 2). These averages are based on very small specimen numbers and hence are only informative.

### (c) Proloculus size

**Figure 4.**
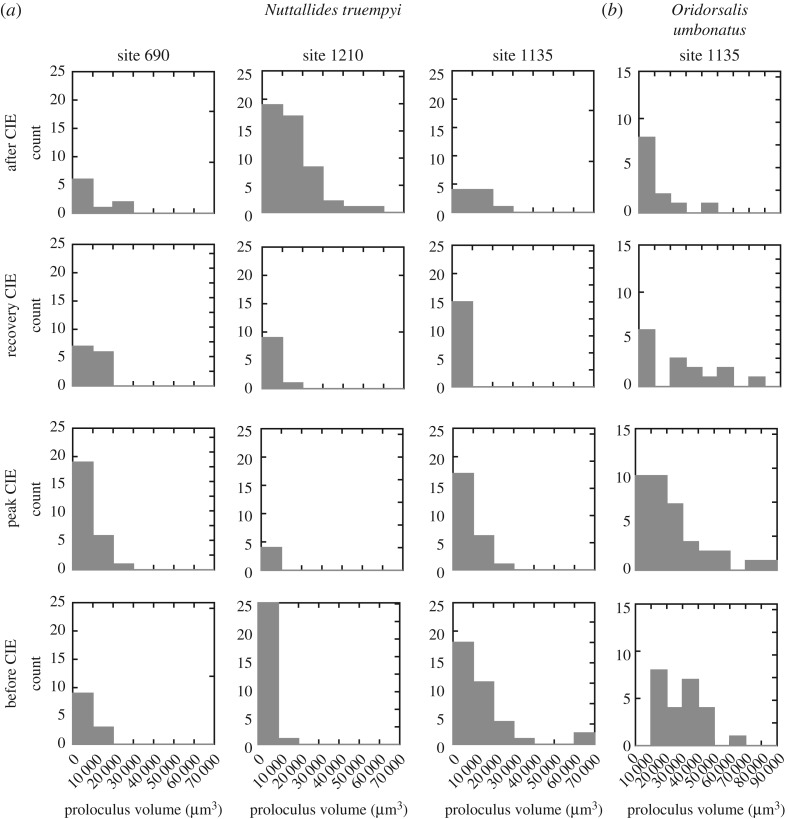
Histogram of proloculus distributions for sites 690, 1210 and 1135 for *N. truempyi* (*a*) and *O. umbonatus* at site 1135 (*b*). Note the difference in scale of the *x*-axis.

Average proloculus sizes for *N. truempyi* range from 150 µm^3^ to approximately 30 000 µm^3^ in all sites, which is equivalent to diameters of 7–40 µm. In a few specimens, the proloculus is larger (electronic supplementary material, figure SI1). At site 1210, *N. truempyi* proloculus sizes are small below and in the peak CIE, with an increase 205 kyr after the event (figure 3*c*). At site 690, small values dominate. Populations at site 1135 show the largest values below the onset of the CIE with a large drop within the CIE (figure 3*c*). Above the CIE, the mean values for each population are within error of each other. None of the *N. truempyi* proloculus size distributions in any of the samples shows a clear bimodality which would allow a clear separation of microspheric and megalospheric forms (figure 4).

*Oridorsalis umbonatus* proloculus are on average two to three times larger than those in *N. truempyi* (1183 µm^3^ to approx. 76 000 µm^3^, equivalent to 40–60 µm diameter), with a clear bimodality before the CIE at site 1135 (figure 4). Unfortunately, we lack data for this species due to dissolution.

### (d) Relationship between size and diameter

One might expect that a larger number of chambers lead to larger size, but the number of chambers is not necessarily a good predictor of the final size in any of the assessed populations, even within a species (electronic supplementary material, figure SI2). Across all sites and time intervals, mean proloculus size and final size chamber within the populations of *N. truempyi* are not significantly correlated, nor is chamber number and final size. By contrast, the mean chamber number and proloculus size of *N. truempyi* are negatively correlated (*r*^2^ = 0.330, *p* = 0.008).

**Figure 5.**
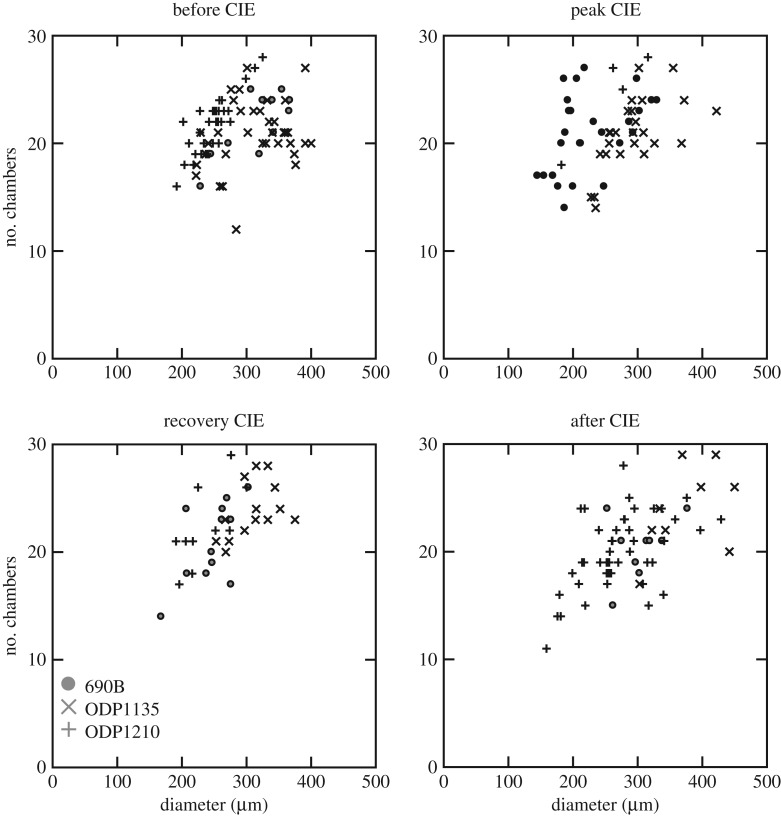
Relationship between final adult diameter and number of chamber for *N. truempyi* for all time slices. Site 690 circles, site 1135× and site 1210 crosses.

In general, population proloculus size and diameter are statistically positively correlated, as small specimens have a small proloculus, whereas large specimens have a proloculus volume above 10 000 µm^3^. At site 1135, a large chamber number results in a large final diameter, though the statistical relationship is not significant due to the small number of specimens. The relation between number of chambers and final diameter appears more strongly controlled for specimens of both species below 250 µm (figure 5). Above 250 µm, a wide range of final sizes can be found at similar chamber numbers, and the same final size can be reached with 15 or 26 chambers, for example. This is also the case for *N. truempyi* at site 1210, whereas there is no clear trend at site 690. The growth trajectories (chambers versus size, figure 5) with time for *N. truempyi* are indistinguishable for all sites. *Oridorsalis umbonatus* at site 1135 adds fewer chambers (electronic supplementary material, figure SI3) than *N. truempyi* to reach the same size in the core CIE and the recovery, but not in the post-CIE.

## 4. Discussion

The main response in morphology during ontogeny of these benthic foraminiferal populations to the environmental changes at the PETM are (i) dwarfing during peak CIE at site 690 to values as low as those at site 1210, (ii) site-specific decreases (site 690), increases (site 1210) or no directional changes in chamber number associated with peak CIE, (iii) low proloculus values at sites 690 and 1210 at peak CIE, associated with a large variability, but no bimodality in the size distribution of the proloculus, and (iv) a lack of relationship between size of the first chamber, number of chambers and final size of the organism across sites and time.

Size is the product of life history of a specimen, including factors such as growth rate (at specific food supply), reproduction and death [60]. Among the physiological factors are metabolic processes such as respiration, ingestion or resistance to starvation, allocation of energy to growth, reproduction or maintenance. As such, dwarfing in benthic foraminifers can be an ecophenotypic response to changes in temperature, oxygen, carbonate ion and food [36]. Within the food web, predator–prey relationships, such as the ability to gather and process prey, become important [61].

Both the absolute temperature and the warming associated with the PETM were similar at our three study sites (figure 2), thus warming *per se* is an unlikely cause for the dwarfism seen at the two deeper sites, but not at the shallowest site 1135. Increased temperatures lead to an increase in food demand, to support the higher metabolic rates. Food probably was most limited in the Pacific gyre (site 1210), where sizes were already small before the CIE, and where some authors suggested (though not quantified) dwarfing in other benthic foraminiferal species than the ones we investigated [39]. This ‘dwarfing’ was largely due to increased abundance of small taxa, not of size changes within survivor taxa. The temporal record of export production for site 690 suggests enhanced remineralization as seen, for example, in the Ba-accumulation rate [48] up to 60 kyr after the onset of the CIE [62], but we do not know whether primary productivity changed, thus whether more or less food reached the benthos. The reduction in size at this site can be interpreted as resource limitation. Small specimens need fewer resources, thus can survive on a smaller amount of food during environmental perturbations impacting food supply. On the other hand, larger size in foraminifers has also been linked to food limitations, i.e. as individuals do not have enough food to reproduce, they keep growing [37]. At site 1210, smaller test size is associated with more chambers (thus a slower rate of increase in test volume if chamber formation occurred at a fixed rate) during the peak CIE, but with fewer chambers at site 690. This observation indicates that the population at site 1210 lived longer while growing more slowly, thus resulting in small size. The other process leading to smaller adult size is accelerated reproduction under optimal conditions (i.e. opportunistic behaviour) as seen, for example, in the short-lived phytodetritus-using living species *Epistominella exigua* (e.g. [59,63]). We would postulate that faster reproduction would lead to a smaller number of chambers, as seen at site 690. A more seasonal food supply (due to its high latitude, thus seasonal darkness), with a temporarily increased food supply resulting in more rapid growth, would result in reproduction at a smaller final test size [38]. Consequently, changes in foraminiferal body size can be the response to both good environmental conditions and stress events.

In our model results, the location of site 690 records the lowest oxygen concentrations of all investigated sites, whereas values at sites 1135 and 1210 were high enough to make physiological responses unlikely. We therefore postulate that the small test size at site 1210 is a combination of low carbonate ion concentrations (figure 2) and low food supply, whereas at site 690 low oxygen availability, possibly in combination with a low food supply, could have resulted in physiological stress.

The tight relation between size and number of chambers in smaller specimens resembles developmental data of planktic foraminifers, which show similar growth trajectories in earlier development, and an increase in plasticity in specimens larger than 100 µm [64]. Increased morphological variability between juveniles and adults has been also documented in other groups such as ammonites, as related to sexual dimorphism in the adult stage [65]. At small sizes, the surface area-to-volume ratio is higher than at larger sizes, facilitating nutrient uptake and diffusion of nutrients, oxygen and carbonate ions. Therefore, small size is preferential during times of reduced oxygen and carbonate ion availability, because of the lower metabolic requirements.

The disadvantage of being small in specimens with asexual reproduction is the lower number of produced offspring. Benthic foraminifers can alternate between sexual and asexual reproduction, thus they could counteract the smaller cytoplasm volume at smaller size by increasing sexual reproduction, if sufficient energy would be available. The use of both reproductive styles would allow the population to respond to environmental conditions by optimizing both energy use and number of offspring. Experiments in larger benthic foraminifers [66] suggest that homeostasis and growth, rather than reproduction, are favoured under stress conditions. Foraminifers mainly die at reproduction, thus continued growth without reproduction may lead to larger sizes.

It is generally asserted that sexual reproduction is indicated by small proloculus sizes at large test diameter, asexual reproduction by large proluculus size, though there are no clear cut-off values for smaller benthic foraminifera in the literature. Analysis of prolocolus sizes in *Uvigerina* species could not corroborate bimodality, but found a correlation between proloculus size and test size [67]. Absolute lower and upper boundaries for proloculus diameter in foraminifera are said to be 4 µm and 1 mm [68]. Given the potential of this method to assess reproductive strategies in the fossil record, it is astonishing how few quantitative data are available on a species level for smaller benthic foraminifera (in contrast with larger benthic foraminifera), and how little is known about environmental drivers of plasticity in both modes of reproduction.

Unexpected outcomes of our study on proloculus sizes are the lack of clear separation between microspheric and megalospheric populations, and the large plasticity in size across the spectrum (see electronic supplementary material, SI1), making our interpretation speculative. The data from the populations at the three sites show different proloculus size changes. Sexual reproduction appears to be favoured at site 1210 up to 63 kyr after the CIE and site 690, whereas site 1135 shows a wide range of proloculus sizes. During the recovery phase of the CIE, both modes of reproduction may have been used at sites 1210 and 690.

The increased complexity of sexual reproduction imposes inherent costs: mates have to be found at low standing stocks, special cell types formed and diploid genomes maintained [69]. In asexual reproduction, every individual has one parent, thus there is no genetic exchange, so that there can be no selection against deleterious mutations [70]. Foraminifers could rapidly change to obligate asexual reproduction if there were no advantage of sexual reproduction. Kondrashov [70] suggested that there is an evolutionary advantage to sexual reproduction in response to ecological changes, by maintaining a better genotype–environment match than possible with asexual reproduction; planktonic foraminifera are described as obligate sexual reproducers [71]. The cost–benefit ratio of sexual versus asexual reproduction may differ radically in different circumstances; for instance, in microorganisms, massive population sizes might be sufficient to avoid the irreversible accumulation of deleterious mutations [72]. The ecological stress, low food, warming, low oxygen and carbonate ion (figure 2) during the PETM might have limited the energy available for sexual reproduction at sites 1210 and 690, thereby increasing the relative proportion of asexual reproduction. In addition, the higher production of offspring by asexual reproduction might be beneficial in seasonal habitats such as the Southern Ocean.

In some samples, two modes of size distribution are tentatively identifiable, but the large range in size, most clearly at site 1135, suggests that other factors than reproductive mode influence proloculus size. Proloculus size has been linked to environmental factors, e.g. a large proloculus at organic pollution (i.e. high food supply) [73], optimal growth and high food availability [74], and temperature and salinity [75,76]. As such, an extensive study of the plasticity of proloculus sizes in modern foraminifers would be timely.

## 5. Conclusion

Our analysis of final size, number of chamber and size of the proloculus of deep-sea benthic foraminifera at three sites shows a highly variable response of morphology to the environmental changes across the PETM. The population at shallowest site 1135 does not show a directional response to the environmental impacts of the PETM, whereas the two other sites record reductions in proloculus size, and at site 690 in overall diameter. Some populations suggest resource limitations, at least seasonally, resulting in small sizes. The driver of this change was not temperature *per se*, nor the increase in temperature, as these were the same at all three sites. We speculate that it was driven by a site-specific combination of food limitations and oxygen changes. Proloculus sizes vary strongly between sites and through time, suggesting a large influence of environment on both microspheric and megalospheric forms without clear bimodality.

## Supplementary Material

Supplementary information

## Data accessibility

The datasets supporting this article have been uploaded onto the web portal www.pangaea.dewww.pangaea.de.
