# Supplementary material for: Strategies in times of crisis—insights into the benthic foraminiferal record of the Palaeocene–Eocene Thermal Maximum
Source: Philos Trans A Math Phys Eng Sci. 2018 Sep 3;376(2130):20170328. doi: 10.1098/rsta.2017.0328 (PMC6127389; doi:10.1098/rsta.2017.0328)
Supplement: Supplementary information [file rsta20170328supp1.docx]

Strategies in times of crisis – insights into the benthic foraminiferal record of the Paleocene Eocene Thermal Maximum

Daniela N Schmidt^1^, Ellen Thomas^2^, Elisabeth Authier^1^, David Sanders^1^, Andy Ridgwell


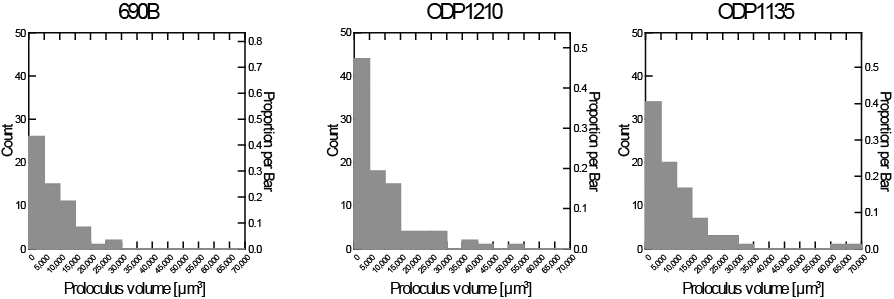


Figure SI 1: Proloculus size for all specimen of *N*. *truempyi* for each Site


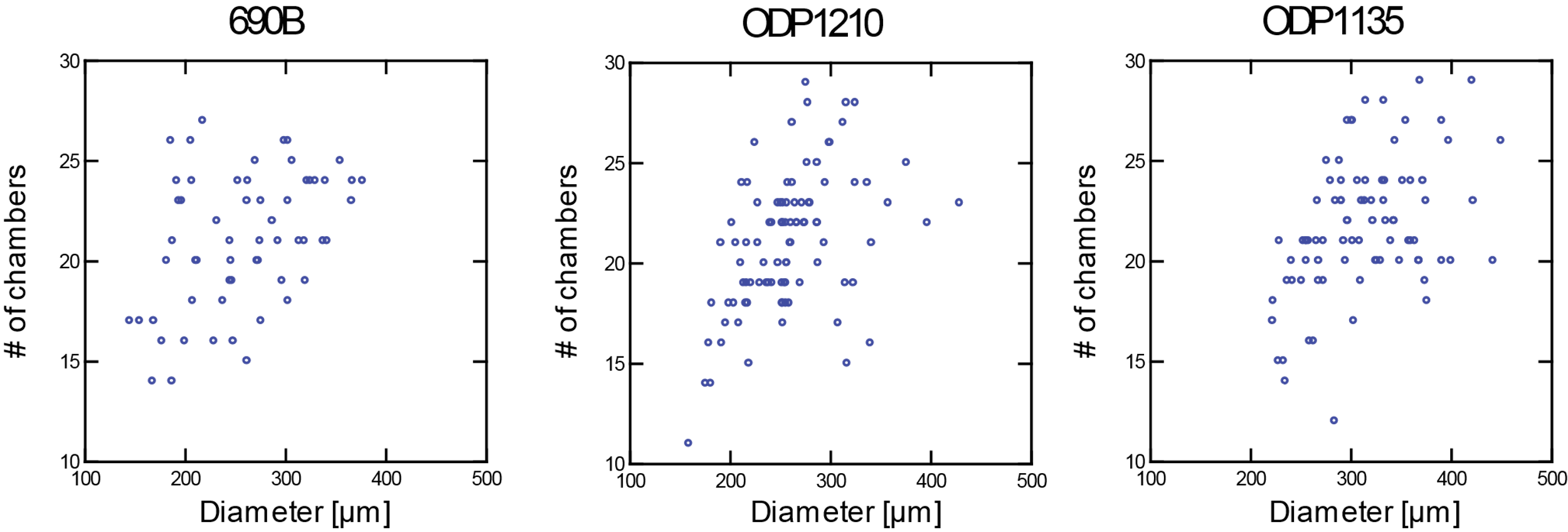


Figure SI 2: Relationship between diameter and chambers for all specimen of *N*. *truempyi* for each Site


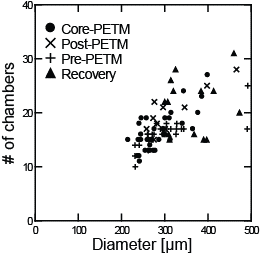


Figure SI3: relationship between diameter and number of chambers for all *Oridorsalis umbonatus* specimen at Site 1135

Table SI 1: indication of preservation determined by numbers (#) of specimen versus specimen with internal dissolution, reported as % (preservation).

|  |  |  | | ***Nuttallides truempyi*** | | ***Oridorsalis umbonatus*** | | |  |
| --- | --- | --- | --- | --- | --- | --- | --- | --- | --- |
| **Site** | **Time (kyr)** | **#** | **# of dissolved specimens** | | **Preservation (%)** | **#** | **# of dissolved specimens** | **Preservation (%)** | |
| 1135 | -1211 | 17 | 5 | | 70.59 | 1 | 0 | 100.00 | |
|  | -35 | 19 | 3 | | 84.21 | 25 | 6 | 76.00 | |
|  | -23 | 7 | 0 | | 100.00 | 6 | 2 | 66.67 | |
|  | 23 | 29 | 3 | | 89.66 | 51 | 14 | 72.55 | |
|  | 140 | 21 | 7 | | 66.67 | 18 | 4 | 77.78 | |
|  | 646 | 9 | 0 | | 100.00 | 17 | 5 | 70.59 | |
| 1210 | -188 | 23 | 0 | | 100.00 | 2 | 0 | 100.00 | |
|  | -62 | 9 | 1 | | 88.89 | 1 | 0 | 100.00 | |
|  | 23 | 4 | 0 | | 100.00 | 1 | 0 | 100.00 | |
|  | 75 | 10 | 0 | | 100.00 | 0 | 0 | ~ | |
|  | 205 | 8 | 0 | | 100.00 | 0 | 0 | ~ | |
|  | 472 | 7 | 0 | | 100.00 | 0 | 0 | ~ | |
| 690B | -19 | 6 | 0 | | 100.00 | 2 | 0 | 100.00 | |
|  | -6 | 6 | 1 | | 83.33 | 0 | 0 | ~ | |
|  | 22 | 12 | 1 | | 91.67 |  |  |  | |
|  | 40 | 16 | 0 | | 100.00 |  |  |  | |
|  | 92 | 7 | 0 | | 100.00 | 4 | 1 | 75.00 | |
|  | 125 | 8 | 3 | | 62.50 | 8 | 4 | 50.00 | |
|  | 655 | 9 | 0 | | 100.00 | 7 | 2 | 71.43 | |

Table SI2: Raw Data of all specimen analysed, core label, time slice the samples was allocated to, age relative to CIE, species (N – *Nuttallides truempyi*, O – *Oridorsalis umbonatus*) proloculus volume, number of chambers and maximum diameter of the specimen. Data is electronically submitted at [www.pangaea.de](http://www.pangaea.de)

| **Sample** | **Time slice** | **Age**  **[kyrs rel CIE]** | **Specie** | **Proloculus volume**  **[µm^3^]** | **Number of chambers** | **Diameter**  **[µm]** |
| --- | --- | --- | --- | --- | --- | --- |
| 1135_26R1_90 | Pre-PETM | -1211.00 | N | 1491.00 | 27.00 | 301.00 |
| 1135_26R1_90 | Pre-PETM | -1211.00 | N | 1455.00 | 25.00 | 276.00 |
| 1135_26R1_90 | Pre-PETM | -1211.00 | N | 18970.00 | 24.00 | 280.00 |
| 1135_26R1_90 | Pre-PETM | -1211.00 | N | 6077.00 | 24.00 | 332.00 |
| 1135_26R1_90 | Pre-PETM | -1211.00 | N | 3365.00 | 23.00 | 311.00 |
| 1135_26R1_90 | Pre-PETM | -1211.00 | N | 9972.00 | 21.00 | 358.00 |
| 1135_26R1_90 | Pre-PETM | -1211.00 | N | 14323.00 | 21.00 | 360.00 |
| 1135_26R1_90 | Pre-PETM | -1211.00 | N | 1368.00 | 21.00 | 229.00 |
| 1135_26R1_90 | Pre-PETM | -1211.00 | N | 14570.00 | 21.00 | 364.00 |
| 1135_26R1_90 | Pre-PETM | -1211.00 | N | 22791.00 | 20.00 | 391.00 |
| 1135_26R1_90 | Pre-PETM | -1211.00 | N | 10453.00 | 20.00 | 349.00 |
| 1135_26R1_90 | Pre-PETM | -1211.00 | N | 8431.00 | 20.00 | 400.00 |
| 1135_25R4_110 | Pre-PETM | -35.00 | N | 6336.00 | 25.00 | 289.00 |
| 1135_25R4_110 | Pre-PETM | -35.00 | N | 11932.00 | 24.00 | 360.00 |
| 1135_25R4_110 | Pre-PETM | -35.00 | N | 29842.00 | 23.00 | 321.00 |
| 1135_25R4_110 | Pre-PETM | -35.00 | N | 2539.00 | 21.00 | 256.00 |
| 1135_25R4_110 | Pre-PETM | -35.00 | N | 2897.00 | 21.00 | 302.00 |
| 1135_25R4_110 | Pre-PETM | -35.00 | N | 12129.00 | 20.00 | 368.00 |
| 1135_25R4_110 | Pre-PETM | -35.00 | N | 28622.00 | 20.00 | 330.00 |
| 1135_25R4_110 | Pre-PETM | -35.00 | N | 3907.00 | 20.00 | 241.00 |
| 1135_25R4_110 | Pre-PETM | -35.00 | N | 2120.00 | 19.00 | 268.00 |
| 1135_25R4_110 | Pre-PETM | -35.00 | N | 4055.00 | 19.00 | 237.00 |
| 1135_25R4_110 | Pre-PETM | -35.00 | N | 7112.00 | 18.00 | 223.00 |
| 1135_25R4_110 | Pre-PETM | -35.00 | N | 66032.00 | 18.00 | 376.00 |
| 1135_25R4_110 | Pre-PETM | -35.00 | N | 13744.00 | 17.00 | 222.00 |
| 1135_25R4_110 | Pre-PETM | -35.00 | N | 33972.00 | 16.00 | 263.00 |
| 1135_25R4_110 | Pre-PETM | -35.00 | N | 18305.00 | 16.00 | 259.00 |
| 1135_25R4_110 | Pre-PETM | -35.00 | N | 62655.00 | 12.00 | 284.00 |
| 1135_25R4_105 | Pre-PETM | -35.00 | N | 2699.00 | 27.00 | 391.00 |
| 1135_25R4_105 | Pre-PETM | -35.00 | N | 17947.00 | 23.00 | 291.00 |
| 1135_25R4_105 | Pre-PETM | -35.00 | N | 5164.00 | 22.00 | 343.00 |
| 1135_25R4_105 | Pre-PETM | -35.00 | N | 16172.00 | 22.00 | 335.00 |
| 1135_25R4_105 | Pre-PETM | -35.00 | N | 22557.00 | 21.00 | 340.00 |
| 1135_25R4_105 | Pre-PETM | -35.00 | N | 1344.00 | 20.00 | 325.00 |
| 1135_25R4_105 | Pre-PETM | -35.00 | N | 10206.00 | 19.00 | 374.00 |
| 1135_25R4_92 | Core-PETM | 23.00 | N | 5399.00 | 27.00 | 355.00 |
| 1135_25R4_92 | Core-PETM | 23.00 | N | 5054.00 | 27.00 | 302.00 |
| 1135_25R4_92 | Core-PETM | 23.00 | N | 9467.00 | 24.00 | 372.00 |
| 1135_25R4_92 | Core-PETM | 23.00 | N | 4401.00 | 24.00 | 307.00 |
| 1135_25R4_92 | Core-PETM | 23.00 | N | 2884.00 | 24.00 | 291.00 |
| 1135_25R4_92 | Core-PETM | 23.00 | N | 1874.00 | 23.00 | 291.00 |
| 1135_25R4_92 | Core-PETM | 23.00 | N | 5226.00 | 23.00 | 285.00 |
| 1135_25R4_92 | Core-PETM | 23.00 | N | 16086.00 | 23.00 | 422.00 |
| 1135_25R4_92 | Core-PETM | 23.00 | N | 1627.00 | 22.00 | 297.00 |
| 1135_25R4_92 | Core-PETM | 23.00 | N | 4043.00 | 21.00 | 309.00 |
| 1135_25R4_92 | Core-PETM | 23.00 | N | 6336.00 | 21.00 | 293.00 |
| 1135_25R4_92 | Core-PETM | 23.00 | N | 13214.00 | 21.00 | 258.00 |
| 1135_25R4_92 | Core-PETM | 23.00 | N | 4068.00 | 21.00 | 256.00 |
| 1135_25R4_92 | Core-PETM | 23.00 | N | 974.00 | 21.00 | 266.00 |
| 1135_25R4_92 | Core-PETM | 23.00 | N | 3932.00 | 20.00 | 256.00 |
| 1135_25R4_92 | Core-PETM | 23.00 | N | 16271.00 | 20.00 | 368.00 |
| 1135_25R4_92 | Core-PETM | 23.00 | N | 9960.00 | 20.00 | 326.00 |
| 1135_25R4_92 | Core-PETM | 23.00 | N | 2958.00 | 20.00 | 295.00 |
| 1135_25R4_92 | Core-PETM | 23.00 | N | 9799.00 | 19.00 | 310.00 |
| 1135_25R4_92 | Core-PETM | 23.00 | N | 12117.00 | 19.00 | 251.00 |
| 1135_25R4_92 | Core-PETM | 23.00 | N | 6755.00 | 19.00 | 242.00 |
| 1135_25R4_92 | Core-PETM | 23.00 | N | 7112.00 | 19.00 | 273.00 |
| 1135_25R4_92 | Core-PETM | 23.00 | N | 12487.00 | 15.00 | 228.00 |
| 1135_25R4_92 | Core-PETM | 23.00 | N | 14767.00 | 15.00 | 233.00 |
| 1135_25R4_92 | Core-PETM | 23.00 | N | 24147.00 | 14.00 | 235.00 |
| 1135_25R4_56 | Recovery | 140.00 | N | 2626.00 |  | 377.00 |
| 1135_25R4_56 | Recovery | 140.00 | N | 2256.00 | 28.00 | 315.00 |
| 1135_25R4_56 | Recovery | 140.00 | N | 4364.00 | 28.00 | 333.00 |
| 1135_25R4_56 | Recovery | 140.00 | N | 2391.00 | 27.00 | 297.00 |
| 1135_25R4_56 | Recovery | 140.00 | N | 3575.00 | 26.00 | 344.00 |
| 1135_25R4_56 | Recovery | 140.00 | N | 5424.00 | 24.00 | 352.00 |
| 1135_25R4_56 | Recovery | 140.00 | N | 1812.00 | 24.00 | 315.00 |
| 1135_25R4_56 | Recovery | 140.00 | N | 2317.00 | 23.00 | 267.00 |
| 1135_25R4_56 | Recovery | 140.00 | N | 5066.00 | 23.00 | 333.00 |
| 1135_25R4_56 | Recovery | 140.00 | N | 2909.00 | 23.00 | 375.00 |
| 1135_25R4_56 | Recovery | 140.00 | N | 6028.00 | 23.00 | 314.00 |
| 1135_25R4_56 | Recovery | 140.00 | N | 4216.00 | 22.00 | 297.00 |
| 1135_25R4_56 | Recovery | 140.00 | N | 7322.00 | 21.00 | 273.00 |
| 1135_25R4_56 | Recovery | 140.00 | N | 2441.00 | 21.00 | 253.00 |
| 1135_25R4_56 | Recovery | 140.00 | N | 3710.00 | 20.00 | 268.00 |
| 1135-25R-3-2 | Post-PETM | 646.00 | N | 4795.00 | 29.00 | 369.00 |
| 1135-25R-3-2 | Post-PETM | 646.00 | N | 6767.00 | 29.00 | 421.00 |
| 1135-25R-3-2 | Post-PETM | 646.00 | N | 14151.00 | 26.00 | 398.00 |
| 1135-25R-3-2 | Post-PETM | 646.00 | N | 19106.00 | 26.00 | 450.00 |
| 1135-25R-3-2 | Post-PETM | 646.00 | N | 3008.00 | 24.00 | 334.00 |
| 1135-25R-3-2 | Post-PETM | 646.00 | N | 25281.00 | 22.00 | 322.00 |
| 1135-25R-3-2 | Post-PETM | 646.00 | N | 3008.00 | 22.00 | 343.00 |
| 1135-25R-3-2 | Post-PETM | 646.00 | N | 14175.00 | 20.00 | 442.00 |
| 1135-25R-3-2 | Post-PETM | 646.00 | N | 11611.00 | 17.00 | 303.00 |
| 1210_20H_6_46 | Recovery | 75.00 | N | 1343.00 | 29.00 | 276.00 |
| 1210_20H_6_46 | Recovery | 75.00 | N | 4721.00 | 26.00 | 300.00 |
| 1210_20H_6_46 | Recovery | 75.00 | N | 764.00 | 26.00 | 225.00 |
| 1210_20H_6_46 | Recovery | 75.00 | N | 3427.00 | 22.00 | 274.00 |
| 1210_20H_6_46 | Recovery | 75.00 | N | 900.00 | 22.00 | 252.00 |
| 1210_20H_6_46 | Recovery | 75.00 | N | 678.00 | 21.00 | 217.00 |
| 1210_20H_6_46 | Recovery | 75.00 | N | 1713.00 | 21.00 | 206.00 |
| 1210_20H_6_46 | Recovery | 75.00 | N | 1282.00 | 21.00 | 191.00 |
| 1210_20H_6_46 | Recovery | 75.00 | N | 12351.00 | 18.00 | 216.00 |
| 1210_20H_6_46 | Recovery | 75.00 | N | 986.00 | 17.00 | 196.00 |
| 1210_20H_6_35 | Post-PETM | 205.00 | N | 13251.00 | 24.00 | 337.00 |
| 1210_20H_6_35 | Post-PETM | 205.00 | N | 1689.00 | 24.00 | 218.00 |
| 1210_20H_6_35 | Post-PETM | 205.00 | N | 370.00 | 24.00 | 212.00 |
| 1210_20H_6_35 | Post-PETM | 205.00 | N | 21534.00 | 22.00 | 397.00 |
| 1210_20H_6_35 | Post-PETM | 205.00 | N | 4844.00 | 19.00 | 217.00 |
| 1210_20H_6_35 | Post-PETM | 205.00 | N | 3192.00 | 18.00 | 199.00 |
| 1210_20H_6_35 | Post-PETM | 205.00 | N | 14619.00 | 17.00 | 308.00 |
| 1210_20H_6_35 | Post-PETM | 205.00 | N | 39740.00 | 15.00 | 317.00 |
| 1210_20H_6_19 | Post-PETM | 472.00 | N | 14385.00 | 23.00 | 429.00 |
| 1210_20H_6_19 | Post-PETM | 472.00 | N | 2995.00 | 22.00 | 240.00 |
| 1210_20H_6_19 | Post-PETM | 472.00 | N | 11809.00 | 21.00 | 341.00 |
| 1210_20H_6_19 | Post-PETM | 472.00 | N | 20819.00 | 20.00 | 288.00 |
| 1210_20H_6_19 | Post-PETM | 472.00 | N | 14927.00 | 19.00 | 315.00 |
| 1210_20H_6_19 | Post-PETM | 472.00 | N | 39099.00 | 19.00 | 323.00 |
| 1210_20H_6_19 | Post-PETM | 472.00 | N | 25933.00 | 16.00 | 340.00 |
| 1209B_21H6_100 | Post-PETM | 967 | N | 21879 | 21 | 294 |
| 1209B_21H6_100 | Post-PETM | 967 | N | 5621 | 16 | 179 |
| 1209B_21H6_100 | Post-PETM | 967 | N | 12893 | 15 | 219 |
| 1209B_21H6_100 | Post-PETM | 967 | N | 7199 | 14 | 176 |
| 1209B_21H6_100 | Post-PETM | 967 | N | 17738 | 19 | 256 |
| 1209B_21H6_100 | Post-PETM | 967 | N | 50156 | 17 | 253 |
| 1209B_21H6_100 | Post-PETM | 967 | N | 43981 | 23 | 358 |
| 1209B_21H6_100 | Post-PETM | 967 | N | 8074 | 19 | 255 |
| 1209B_21H6_100 | Post-PETM | 967 | N | 14668 | 18 | 252 |
| 1209B_21H6_100 | Post-PETM | 967 | N |  |  | 273 |
| 1209B_21H6_100 | Post-PETM | 967 | N | 12178 | 19 | 252 |
| 1209B_21H6_100 | Post-PETM | 967 | N | 15100 | 11 | 159 |
| 1209B_21H6_100 | Post-PETM | 967 | N | 26909 | 25 | 376 |
| 1209B_21H6_100 | Post-PETM | 967 | N | 2502 | 24 | 295 |
| 1209B_21H6_100 | Post-PETM | 967 | N | 5658 | 23 | 280 |
| 1209B_21H6_100 | Post-PETM | 967 | N | 9109 | 17 | 209 |
| 1209B_21H6_100 | Post-PETM | 967 | N | 27217 | 21 | 260 |
| 1209B_21H6_100 | Post-PETM | 967 | N | 21140 | 18 | 256 |
| 1209B_21H6_100 | Post-PETM | 967 | N | 2502 | 28 | 278 |
| 1209B_21H6_100 | Post-PETM | 967 | N | 5448 | 19 | 214 |
| 1209B_21H6_100 | Post-PETM | 967 | N | 14619 | 22 | 287 |
| 1209B_21H6_100 | Post-PETM | 967 | N | 28597 | 18 | 253 |
| 1209B_21H6_100 | Post-PETM | 967 | N | 14792 | 25 | 287 |
| 1209B_21H6_100 | Post-PETM | 967 | N | 8641 | 14 | 181 |
| 1209B_21H6_100 | Post-PETM | 967 | N | 11550 | 19 | 270 |
| 1209B_21H6_100 | Post-PETM | 967 | N | 2478 | 24 | 325 |
| 1209B_21H6_100 | Post-PETM | 967 | N | 9232 | 19 | 242 |
| 1209B_21H6_100 | Post-PETM | 967 | N | 18314 | 18 | 259 |
| 1209B_21H6_100 | Post-PETM | 967 | N | 6681 | 20 | 257 |
| 1209B_21H6_100 | Post-PETM | 967 | N | 11020 | 21 | 261 |
| 1209B_21H6_100 | Post-PETM | 967 | N | 19365 | 22 | 287 |
| 1209B_21H6_100 | Post-PETM | 967 | N | 13670 | 22 | 267 |
| 1209B_21H6_100 | Post-PETM | 967 | N | 7642 | 22 | 267 |
| 1209B_21H6_100 | Post-PETM | 967 | N | 9171 | 23 | 279 |
| 1210_20H_6_62 | Pre-PETM | -188.00 | N | 11266.00 | 28.00 | 325.00 |
| 1210_20H_6_62 | Pre-PETM | -188.00 | N | 1652.00 | 26.00 | 299.00 |
| 1210_20H_6_62 | Pre-PETM | -188.00 | N | 2539.00 | 24.00 | 262.00 |
| 1210_20H_6_62 | Pre-PETM | -188.00 | N | 7051.00 | 23.00 | 251.00 |
| 1210_20H_6_62 | Pre-PETM | -188.00 | N | 1381.00 | 23.00 | 253.00 |
| 1210_20H_6_62 | Pre-PETM | -188.00 | N | 3131.00 | 23.00 | 265.00 |
| 1210_20H_6_62 | Pre-PETM | -188.00 | N | 2650.00 | 23.00 | 248.00 |
| 1210_20H_6_62 | Pre-PETM | -188.00 | N | 4795.00 | 23.00 | 272.00 |
| 1210_20H_6_62 | Pre-PETM | -188.00 | N | 3488.00 | 22.00 | 253.00 |
| 1210_20H_6_62 | Pre-PETM | -188.00 | N | 1368.00 | 22.00 | 253.00 |
| 1210_20H_6_62 | Pre-PETM | -188.00 | N | 9467.00 | 22.00 | 275.00 |
| 1210_20H_6_62 | Pre-PETM | -188.00 | N | 5892.00 | 22.00 | 261.00 |
| 1210_20H_6_62 | Pre-PETM | -188.00 | N | 1849.00 | 22.00 | 242.00 |
| 1210_20H_6_62 | Pre-PETM | -188.00 | N | 4881.00 | 21.00 | 228.00 |
| 1210_20H_6_62 | Pre-PETM | -188.00 | N | 6447.00 | 20.00 | 248.00 |
| 1210_20H_6_62 | Pre-PETM | -188.00 | N | 2626.00 | 20.00 | 234.00 |
| 1210_20H_6_62 | Pre-PETM | -188.00 | N | 3254.00 | 20.00 | 257.00 |
| 1210_20H_6_62 | Pre-PETM | -188.00 | N | 3242.00 | 19.00 | 221.00 |
| 1210_20H_6_62 | Pre-PETM | -188.00 | N | 8407.00 | 19.00 | 230.00 |
| 1210_20H_6_62 | Pre-PETM | -188.00 | N | 4857.00 | 19.00 | 239.00 |
| 1210_20H_6_62 | Pre-PETM | -188.00 | N | 4795.00 | 19.00 | 237.00 |
| 1210_20H_6_62 | Pre-PETM | -188.00 | N | 8172.00 | 18.00 | 218.00 |
| 1210_20H_6_62 | Pre-PETM | -188.00 | N | 6249.00 | 16.00 | 192.00 |
| 1210_20H_6_55 | Pre-PETM | -62.00 | N | 949.00 | 27.00 | 313.00 |
| 1210_20H_6_55 | Pre-PETM | -62.00 | N | 2970.00 | 24.00 | 258.00 |
| 1210_20H_6_55 | Pre-PETM | -62.00 | N | 2157.00 | 23.00 | 228.00 |
| 1210_20H_6_55 | Pre-PETM | -62.00 | N | 1442.00 | 23.00 | 257.00 |
| 1210_20H_6_55 | Pre-PETM | -62.00 | N | 3131.00 | 22.00 | 256.00 |
| 1210_20H_6_55 | Pre-PETM | -62.00 | N | 1911.00 | 22.00 | 202.00 |
| 1210_20H_6_55 | Pre-PETM | -62.00 | N | 1787.00 | 20.00 | 211.00 |
| 1210_20H_6_55 | Pre-PETM | -62.00 | N | 776.00 | 18.00 | 204.00 |
| 1210_20H_6_50 | Core-PETM | 23.00 | N | 1738.00 | 28.00 | 316.00 |
| 1210_20H_6_50 | Core-PETM | 23.00 | N | 2095.00 | 27.00 | 262.00 |
| 1210_20H_6_50 | Core-PETM | 23.00 | N | 752.00 | 25.00 | 277.00 |
| 1210_20H_6_50 | Core-PETM | 23.00 | N | 1270.00 | 18.00 | 182.00 |
| 690B-19H-3-118 | Pre-PETM | -19.00 | N | 2071.00 | 25.00 | 307.00 |
| 690B-19H-3-118 | Pre-PETM | -19.00 | N | 3069.00 | 24.00 | 340.00 |
| 690B-19H-3-118 | Pre-PETM | -19.00 | N | 5880.00 | 24.00 | 367.00 |
| 690B-19H-3-118 | Pre-PETM | -19.00 | N | 4943.00 | 24.00 | 325.00 |
| 690B-19H-3-118 | Pre-PETM | -19.00 | N | 13497.00 | 21.00 | 342.00 |
| 690B-19H-3-118 | Pre-PETM | -19.00 | N | 16653.00 | 19.00 | 320.00 |
| 690B-19H-3-86 | Pre-PETM | -19.00 | N | 1516.00 |  | 274.00 |
| 690B-19H-3-86 | Pre-PETM | -19.00 | N | 13510.00 | 25.00 | 355.00 |
| 690B-19H-3-86 | Pre-PETM | -19.00 | N | 7568.00 | 23.00 | 366.00 |
| 690B-19H-3-86 | Pre-PETM | -19.00 | N | 4881.00 | 20.00 | 272.00 |
| 690B-19H-3-86 | Pre-PETM | -19.00 | N | 3427.00 | 19.00 | 245.00 |
| 690B-19H-3-86 | Pre-PETM | -19.00 | N | 3981.00 | 16.00 | 229.00 |
| 690B-19H3-43-44 | Core-PETM | 22 | N | 15297 | 22 | 232 |
| 690B-19H3-43-44 | Core-PETM | 22 | N | 1972 | 26 | 299 |
| 690B-19H3-43-44 | Core-PETM | 22 | N | 9528 | 20 | 273 |
| 690B-19H3-43-44 | Core-PETM | 22 | N | 1196 | 26 | 186 |
| 690B-19H3-43-44 | Core-PETM | 22 | N | 2860 | 20 | 211 |
| 690B-19H3-43-44 | Core-PETM | 22 | N | 1171 | 26 | 206 |
| 690B-19H3-43-44 | Core-PETM | 22 | N | 8185 | 20 | 212 |
| 690B-19H3-43-44 | Core-PETM | 22 | N | 7655 | 23 | 303 |
| 690B-19H3-43-44 | Core-PETM | 22 | N | 11538 | 24 | 322 |
| 690B-19H3-43-44 | Core-PETM | 22 | N | 5473 | 17 | 145 |
| 690B-19H3-43-44 | Core-PETM | 22 | N | 1159 | 24 | 192 |
| 690B-19H3-43-44 | Core-PETM | 22 | N | 4548 | 14 | 187 |
| 690B-19H3-43-44 | Core-PETM | 22 | N | 6533 | 16 | 177 |
| 690B-19H3-43-44 | Core-PETM | 22 | N | 715 | 21 | 188 |
| 690B-19H3-43-44 | Core-PETM | 22 | N | 1689 | 27 | 218 |
| 690B-19H3-43-44 | Core-PETM | 22 | N | 4746 | 23 | 194 |
| 690B-19H3-15-16 | Core-PETM | 40 | N | 2194 | 23 | 197 |
| 690B-19H3-15-16 | Core-PETM | 40 | N | 7149 | 21 | 245 |
| 690B-19H3-15-16 | Core-PETM | 40 | N | 12832 | 16 | 200 |
| 690B-19H3-15-16 | Core-PETM | 40 | N | 21534 | 16 | 248 |
| 690B-19H3-15-16 | Core-PETM | 40 | N | 12289 | 21 | 293 |
| 690B-19H3-15-16 | Core-PETM | 40 | N | 4277 | 20 | 182 |
| 690B-19H3-15-16 | Core-PETM | 40 | N | 10219 | 22 | 287 |
| 690B-19H3-15-16 | Core-PETM | 40 | N | 3476 | 17 | 155 |
| 690B-19H3-15-16 | Core-PETM | 40 | N | 12709 | 24 | 330 |
| 690B-19H3-15-16 | Core-PETM | 40 | N |  |  | 137 |
| 690B-19H3-15-16 | Core-PETM | 40 | N | 9997 | 17 | 169 |
| 690B-19H-2-77 | Recovery | 92.00 | N | 481.00 | 24.00 | 207.00 |
| 690B-19H-2-77 | Recovery | 91.00 | N | 4216.00 | 23.00 | 262.00 |
| 690B-19H-2-77 | Recovery | 92.00 | N | 11587.00 | 20.00 | 246.00 |
| 690B-19H-2-77 | Recovery | 92.00 | N | 11339.00 | 18.00 | 238.00 |
| 690B-19H-2-77 | Recovery | 92.00 | N | 3045.00 | 18.00 | 208.00 |
| 690B-19H-2-77 | Recovery | 92.00 | N | 15963.00 | 17.00 | 276.00 |
| 690B-19H-2-77 | Recovery | 92.00 | N | 13916.00 | 14.00 | 168.00 |
| 690B-19H-1-114 | Recovery | 125.00 | N | 1837.00 |  | 307.00 |
| 690B-19H-1-114 | Recovery | 125.00 | N | 14336.00 | 26.00 | 303.00 |
| 690B-19H-1-114 | Recovery | 125.00 | N | 4746.00 | 25.00 | 270.00 |
| 690B-19H-1-114 | Recovery | 125.00 | N | 7815.00 | 24.00 | 263.00 |
| 690B-19H-1-114 | Recovery | 125.00 | N | 9898.00 | 23.00 | 276.00 |
| 690B-19H-1-114 | Recovery | 125.00 | N | 19192.00 | 19.00 | 247.00 |
| 690B-17H_3_74 | Post-PETM | 655.00 | N | 28930.00 | 24.00 | 377.00 |
| 690B-17H_3_74 | Post-PETM | 655.00 | N | 1393.00 | 24.00 | 253.00 |
| 690B-17H_3_74 | Post-PETM | 655.00 | N | 17134.00 | 21.00 | 338.00 |
| 690B-17H_3_74 | Post-PETM | 655.00 | N | 9035.00 | 21.00 | 275.00 |
| 690B-17H_3_74 | Post-PETM | 655.00 | N | 6114.00 | 21.00 | 314.00 |
| 690B-17H_3_74 | Post-PETM | 655.00 | N | 3907.00 | 21.00 | 319.00 |
| 690B-17H_3_74 | Post-PETM | 655.00 | N | 8826.00 | 19.00 | 297.00 |
| 690B-17H_3_74 | Post-PETM | 655.00 | N | 8764.00 | 18.00 | 303.00 |
| 690B-17H_3_74 | Post-PETM | 655.00 | N | 28548.00 | 15.00 | 262.00 |
|  |  |  |  |  |  |  |
| 1135_25R4_110 | Pre-PETM | -35.00 | O | 16061.00 | 25.00 | 492.00 |
| 1135_25R4_110 | Pre-PETM | -35.00 | O | 22668.00 | 18.00 | 281.00 |
| 1135_25R4_110 | Pre-PETM | -35.00 | O | 18107.00 | 18.00 | 304.00 |
| 1135_25R4_110 | Pre-PETM | -35.00 | O | 61361.00 | 17.00 | 344.00 |
| 1135_25R4_110 | Pre-PETM | -35.00 | O | 31630.00 | 17.00 | 314.00 |
| 1135_25R4_110 | Pre-PETM | -35.00 | O | 33158.00 | 17.00 | 290.00 |
| 1135_25R4_110 | Pre-PETM | -35.00 | O | 14237.00 | 17.00 | 303.00 |
| 1135_25R4_110 | Pre-PETM | -35.00 | O | 36782.00 | 16.00 | 326.00 |
| 1135_25R4_110 | Pre-PETM | -35.00 | O | 18909.00 | 16.00 | 298.00 |
| 1135_25R4_110 | Pre-PETM | -35.00 | O | 11316.00 | 16.00 | 300.00 |
| 1135_25R4_110 | Pre-PETM | -35.00 | O | 15852.00 | 16.00 | 261.00 |
| 1135_25R4_110 | Pre-PETM | -35.00 | O | 38027.00 | 16.00 | 275.00 |
| 1135_25R4_110 | Pre-PETM | -35.00 | O | 18983.00 | 16.00 | 260.00 |
| 1135_25R4_110 | Pre-PETM | -35.00 | O | 35303.00 | 16.00 | 271.00 |
| 1135_25R4_110 | Pre-PETM | -35.00 | O | 44856.00 | 14.00 | 231.00 |
| 1135_25R4_110 | Pre-PETM | -35.00 | O | 10601.00 | 14.00 | 241.00 |
| 1135_25R4_110 | Pre-PETM | -35.00 | O | 45065.00 | 13.00 | 262.00 |
| 1135_25R4_110 | Pre-PETM | -35.00 | O | 38483.00 | 12.00 | 231.00 |
| 1135_25R4_110 | Pre-PETM | -35.00 | O | 37743.00 | 10.00 | 232.00 |
| 1135_25R4_105 | Pre-PETM | -23.00 | O | 28301.00 | 18.00 | 328.00 |
| 1135_25R4_105 | Pre-PETM | -23.00 | O | 46520.00 | 17.00 | 491.00 |
| 1135_25R4_105 | Pre-PETM | -23.00 | O | 29250.00 | 17.00 | 339.00 |
| 1135_25R4_105 | Pre-PETM | -23.00 | O | 22052.00 | 16.00 | 294.00 |
| 1135_25R4_92 | Core-PETM | 23.00 | O | 1368.00 | 27.00 | 399.00 |
| 1135_25R4_92 | Core-PETM | 23.00 | O | 2083.00 | 24.00 | 344.00 |
| 1135_25R4_92 | Core-PETM | 23.00 | O | 12561.00 | 23.00 | 386.00 |
| 1135_25R4_92 | Core-PETM | 23.00 | O | 2416.00 | 20.00 | 315.00 |
| 1135_25R4_92 | Core-PETM | 23.00 | O | 6595.00 | 20.00 | 377.00 |
| 1135_25R4_92 | Core-PETM | 23.00 | O | 986.00 | 19.00 | 258.00 |
| 1135_25R4_92 | Core-PETM | 23.00 | O | 2490.00 | 19.00 | 246.00 |
| 1135_25R4_92 | Core-PETM | 23.00 | O | 15741.00 | 19.00 | 311.00 |
| 1135_25R4_92 | Core-PETM | 23.00 | O | 1282.00 | 19.00 | 301.00 |
| 1135_25R4_92 | Core-PETM | 23.00 | O | 3612.00 | 18.00 | 341.00 |
| 1135_25R4_92 | Core-PETM | 23.00 | O | 7223.00 | 18.00 | 241.00 |
| 1135_25R4_92 | Core-PETM | 23.00 | O | 10699.00 | 17.00 | 276.00 |
| 1135_25R4_92 | Core-PETM | 23.00 | O | 28375.00 | 17.00 | 297.00 |
| 1135_25R4_92 | Core-PETM | 23.00 | O | 2342.00 | 17.00 | 297.00 |
| 1135_25R4_92 | Core-PETM | 23.00 | O | 26650.00 | 17.00 | 365.00 |
| 1135_25R4_92 | Core-PETM | 23.00 | O | 36659.00 | 16.00 | 300.00 |
| 1135_25R4_92 | Core-PETM | 23.00 | O | 20117.00 | 16.00 | 298.00 |
| 1135_25R4_92 | Core-PETM | 23.00 | O | 13707.00 | 16.00 | 245.00 |
| 1135_25R4_92 | Core-PETM | 23.00 | O | 30261.00 | 16.00 | 300.00 |
| 1135_25R4_92 | Core-PETM | 23.00 | O | 13818.00 | 15.00 | 239.00 |
| 1135_25R4_92 | Core-PETM | 23.00 | O | 10761.00 | 15.00 | 215.00 |
| 1135_25R4_92 | Core-PETM | 23.00 | O | 29891.00 | 15.00 | 268.00 |
| 1135_25R4_92 | Core-PETM | 23.00 | O | 34539.00 | 15.00 | 269.00 |
| 1135_25R4_92 | Core-PETM | 23.00 | O | 20080.00 | 15.00 | 287.00 |
| 1135_25R4_92 | Core-PETM | 23.00 | O | 15839.00 | 15.00 | 260.00 |
| 1135_25R4_92 | Core-PETM | 23.00 | O | 12203.00 | 15.00 | 245.00 |
| 1135_25R4_92 | Core-PETM | 23.00 | O | 17466.00 | 15.00 | 313.00 |
| 1135_25R4_92 | Core-PETM | 23.00 | O | 29978.00 | 14.00 | 266.00 |
| 1135_25R4_92 | Core-PETM | 23.00 | O | 41035.00 | 13.00 | 263.00 |
| 1135_25R4_92 | Core-PETM | 23.00 | O | 52942.00 | 13.00 | 274.00 |
| 1135_25R4_92 | Core-PETM | 23.00 | O | 72171.00 | 13.00 | 266.00 |
| 1135_25R4_92 | Core-PETM | 23.00 | O | 83807.00 | 13.00 | 278.00 |
| 1135_25R4_92 | Core-PETM | 23.00 | O | 19661.00 | 13.00 | 255.00 |
| 1135_25R4_92 | Core-PETM | 23.00 | O | 41823.00 | 12.00 | 241.00 |
| 1135_25R4_92 | Core-PETM | 23.00 | O | 26872.00 | 12.00 | 237.00 |
| 1135_25R4_92 | Core-PETM | 23.00 | O | 59376.00 | 11.00 | 242.00 |
| 1135_25R4_56 | Recovery | 140.00 | O | 22434.00 |  | 328.00 |
| 1135_25R4_56 | Recovery | 140.00 | O | 6065.00 | 31.00 | 460.00 |
| 1135_25R4_56 | Recovery | 140.00 | O | 5830.00 | 28.00 | 325.00 |
| 1135_25R4_56 | Recovery | 140.00 | O | 1294.00 | 26.00 | 314.00 |
| 1135_25R4_56 | Recovery | 140.00 | O | 24000.00 | 24.00 | 413.00 |
| 1135_25R4_56 | Recovery | 140.00 | O | 5000.00 | 24.00 | 320.00 |
| 1135_25R4_56 | Recovery | 140.00 | O | 46298.00 | 24.00 | 384.00 |
| 1135_25R4_56 | Recovery | 140.00 | O | 6558.00 | 22.00 | 307.00 |
| 1135_25R4_56 | Recovery | 140.00 | O | 5000.00 | 22.00 | 300.00 |
| 1135_25R4_56 | Recovery | 140.00 | O | 52153.00 | 20.00 | 473.00 |
| 1135_25R4_56 | Recovery | 140.00 | O | 32961.00 | 16.00 | 309.00 |
| 1135_25R4_56 | Recovery | 140.00 | O | 76424.00 | 16.00 | 368.00 |
| 1135_25R4_56 | Recovery | 140.00 | O | 37608.00 | 15.00 | 390.00 |
| 1135_25R4_56 | Recovery | 140.00 | O | 21140.00 | 15.00 | 397.00 |
| 1135_25R4_56 | Recovery | 140.00 | O | 50020.00 | 15.00 | 311.00 |
| 1135-25R-3-2 | Post-PETM | 646.00 | O | 45201.00 |  | 308.00 |
| 1135-25R-3-2 | Post-PETM | 646.00 | O | 3427.00 | 28.00 | 466.00 |
| 1135-25R-3-2 | Post-PETM | 646.00 | O | 3266.00 | 25.00 | 398.00 |
| 1135-25R-3-2 | Post-PETM | 646.00 | O | 4696.00 | 22.00 | 276.00 |
| 1135-25R-3-2 | Post-PETM | 646.00 | O | 9849.00 | 21.00 | 296.00 |
| 1135-25R-3-2 | Post-PETM | 646.00 | O | 5263.00 | 21.00 | 346.00 |
| 1135-25R-3-2 | Post-PETM | 646.00 | O | 7334.00 | 19.00 | 273.00 |
| 1135-25R-3-2 | Post-PETM | 646.00 | O | 10699.00 | 18.00 | 282.00 |
| 1135-25R-3-2 | Post-PETM | 646.00 | O | 2613.00 | 17.00 | 258.00 |
| 1135-25R-3-2 | Post-PETM | 646.00 | O | 1911.00 | 17.00 | 301.00 |
| 1135-25R-3-2 | Post-PETM | 646.00 | O | 25935.00 | 15.00 | 275.00 |
| 1135-25R-3-2 | Post-PETM | 646.00 | O | 11032.00 | 15.00 | 268.00 |
| 1135_26R1_90 | Pre-PETM | -1211.00 | O | 42662.00 | 17.00 | 327.00 |
| 690B-19H-3-118 | Pre-PETM | -19.00 | O | 17713.00 | 21.00 | 379.00 |
| 690B-19H-3-118 | Pre-PETM | -19.00 | O | 29583.00 | 18.00 | 401.00 |
| 690B-19H3-15-16 | Core-PETM | 40 | O | 49614 |  | 404 |
| 690B-19H-2-77 | Recovery | 92.00 | O | 9035.00 | 24.00 | 296.00 |
| 690B-19H-2-77 | Recovery | 92.00 | O | 13966.00 | 23.00 | 261.00 |
| 690B-19H-2-77 | Recovery | 92.00 | O | 8271.00 | 20.00 | 213.00 |
| 690B-19H-1-114 | Recovery | 125.00 | O | 789.00 | 22.00 | 273.00 |
| 690B-19H-1-114 | Recovery | 125.00 | O | 36942.00 | 15.00 | 310.00 |
| 690B-19H-1-114 | Recovery | 125.00 | O | 44893.00 | 13.00 | 266.00 |
| 690B-19H-1-114 | Recovery | 125.00 | O | 42304.00 | 13.00 | 253.00 |
| 690B-17H_3_74 | Post-PETM | 652.70 | O | 30212.00 |  | 264.00 |
| 690B-17H_3_74 | Post-PETM | 652.70 | O | 1911.00 | 26.00 | 423.00 |
| 690B-17H_3_74 | Post-PETM | 652.70 | O | 1183.00 | 21.00 | 292.00 |
| 690B-17H_3_74 | Post-PETM | 652.70 | O | 59216.00 | 15.00 | 319.00 |
| 690B-17H_3_74 | Post-PETM | 652.70 | O | 21645.00 | 14.00 | 262.00 |
| 690B-17H_3_74 | Post-PETM | 652.70 | O | 41022.00 | 12.00 | 270.00 |
| 1210_20H_6_62 | Pre-PETM | -188.00 | O | 2786.00 | 22.00 | 209.00 |
| 1210_20H_6_62 | Pre-PETM | -188.00 | O | 38224.00 | 13.00 | 223.00 |
| 1210_20H_6_55 | Pre-PETM | -62.00 | O | 12980.00 |  | 407.00 |
| 1210_20H_6_55 | Pre-PETM | -62.00 | O | 14373.00 | 18.00 | 286.00 |
| 1210_20H_6_55 | Pre-PETM | -62.00 | O | 20523.00 | 10.00 | 248.00 |
| 1210_20H_6_50 | Core-PETM | 23.00 | O | 31112.00 | 17.00 | 332.00 |
